# A theory of change of an innovation for therapeutic care and meaningful living in a German nursing home

**DOI:** 10.1186/s12877-022-03462-0

**Published:** 2022-11-11

**Authors:** Anke Desch, Bernd Förstner, Jörg Artmann, Andreas Häusler, Michael Hauptmann, Sibel Altin, Michael Rapp, Christine Holmberg

**Affiliations:** 1 Institute of Social Medicine and Epidemiology, Brandenburg Medical School Theodor Fontane, Brandenburg an der Havel, Germany; 2 Social and Preventive Medicine, Department of Sports and Health Sciences, University of Potsdam, Potsdam, Germany; 3 AOK Rhineland/Hamburg, Düsseldorf, Germany; 4 Institute of Biostatistics and Registry Research, Brandenburg Medical School Theodor Fontane, Brandenburg an der Havel, Germany

**Keywords:** Positive gerontology, De-institutionalization, Meaning-making, Therapeutic emplotment, Integrated narrative nursing, Nursing home culture change

## Abstract

**Background:**

Demographic changes are leading to growing care needs of older people and creating a challenge for healthcare systems worldwide. Nursing homes (NHs) need to provide care for growing numbers of residents while ensuring a high-quality care. We aimed to examine an innovative NH in Germany and apply a theory of change (ToC) approach to develop a best practice model (BPM) for therapeutic care in NHs.

**Methods:**

A multimethod qualitative study conducted from February to July 2021 in Germany involved interviews with 14 staff members of an innovative NH and 10 directors and care managers of other NHs. The interview guidelines included questions on nursing practices, infrastructure, resources, interprofessional collaboration, and working culture. Additional material on the participating NH (website, promotion videos, newsletters, care documentation) were collected. Contextual literature on NH culture and therapeutic care in Germany, ToC methodology, and NH culture change were reviewed. Following a question-focused analysis of all material, we generated a ToC model towards a BPM of therapeutic care and meaningful living in NHs. Results were verified in interdisciplinary team meetings, with study participants and other stakeholders to establish consensus.

**Results:**

The participating NH’s care concept aims to improve residents’ functional abilities and wellbeing as well as staff members’ job satisfaction. Central components of their approach include therapeutic elements such as music and movement in all nursing activities, multidisciplinary collaboration, a broad therapy and social activity offer, the continuation of therapy in everyday activities, a focus on individual life history, values, needs, and skills, social integration into the regional community, and the creation of a meaningful living environment for residents and staff.

**Conclusion:**

The BPM we developed shows how a meaningful living environment can be created through therapeutic care and integrative activities. The ToC sheds light onto the contextual factors and cultural values which should be considered in the development of NH interventions. Research on not only biomedical aspects, but also psychosocial dynamics and narrative co-constructions in nursing practice should inform NH innovations. The ToC also highlights the importance of developing adequate political frameworks and infrastructures for implementing such innovative practices on a larger scale.

## Background

Health care systems around the world are facing substantial challenges to meet the increasing demand of care for older people. Demographic changes such as a higher life expectancy along with multimorbidity in old age contribute to rapidly growing care needs [1, 2]. Globally, the number of people over 60 years is expected to rise from 11 to 22% between 2015 and 2050 [3]. In Germany, the amount of people in need for home or stationary care (“Pflegebedürftigkeit”, divided into five levels ranging from minor to substantial support needs due to mental or physical impairments) more than doubled from 2.02 to 4.13 million between 1999 and 2019 [4]. While most older adults received care in their own home (by either relatives or mobile nursing services), around 800,000 people required admission to nursing homes (NHs) [4]. NHs in Germany are run by either non-profit organizations (52.8%), for-profit companies (42.7%), or by communal organizations or other public institutions [4]. Funding for NH care is partially covered by the statutory nursing care insurance. Remaining costs need to be covered by the residents and their families. During the past fifteen years, the number of NH residents in Germany increased by 24.5% [4].

Health systems are not only facing the task of meeting these growing care demands of older people, but also of ensuring a high quality care that recognizes NH residents’ physiological, psychological and social care needs [5]. Incomplete medical or psychosocial care is a common problem in NHs due to different organizational shortcomings such as poor infrastructure (i.e., equipment, facilities), management problems, or inadequate staff levels [6, 7]. Many older adults experience multimorbidity which contributes to a subsequent loss of autonomy, life-satisfaction, and self-sufficiency [8]. Moreover, studies show that between 10 and 29% of NH residents experience depression and around 58% are diagnosed with dementia [9]. Causes for depression can be attributed to medical conditions and a range of biopsychosocial factors such as the reduced ability to manage one’s living environment, a perceived lack of support from others, or loss of purpose in life [10].

Compared to other countries such as the USA, Canada, Australia, or the Netherlands where advanced practice nurses (APNs) or nurse practitioners (NPs) are well established, the role of nurses remains limited in Germany. The concept of APNs has been introduced to the German healthcare system since the early 2000s, yet, there remains a lack of clarity concerning the legal definition and responsibility of the role [11]. Highly specialized but fragmented healthcare structures and hierarchical interprofessional relationships (especially physicians’ monopoly position) compound the development of clarity towards the APN role [12]. Currently, APNs mainly work in management positions or special care units where they have less direct patient interaction than in other clinical settings [12].

To reduce patients’ care needs and improve their quality of life, nursing concepts of activating, therapeutic, or rehabilitative care have been developed [13–19]. While definitions vary, these concepts share aspects towards promoting patient mobility or functionality, self-care competences, and social integration [17]. “Activating care” seeks to motivate patients to engage in basic care activities and improve their self-sufficiency, but does not explicitly define a therapeutic or rehabilitative goal [15]. “Therapeutic care” aims to restore specific competences by incorporating therapeutic elements into daily care practices [13, 14]. The delivery of therapeutic care is underpinned by the principles of empathy and fostering positive relationships that promote a patients’ physical and emotional wellbeing and medical treatment success [19]. “Rehabilitative care” follows a specific rehabilitation goal and timeframe and is accompanied by a multiprofessional team who works on restoring patients’ competences in a specific domain [16, 18].

While activating care is claimed as a general nursing standard in most German clinics and NHs, therapeutic or rehabilitative care is not yet systematically practiced. According to a study on Berlin NHs by Garms-Homolová and Theiss, activating care practices are not applied effectively, whereby residents did not receive enough activation or were included despite cognitive impairments due to which they were unable to follow the activating encouragements effectively [15, 20]. Results from a qualitative study by Lautenschläger indicate that even in rehabilitation clinics, nurses’ knowledge and conduct of therapeutic care has been shown to vary widely and further training is recommended [21]. In another case study, Lautenschläger, Muser, Müller and other members of their research team provide a description of how the theory of therapeutic care can be transferred into neurologic nursing practice [22].Thus far, studies exploring the application of therapeutic or rehabilitative care in German NHs (especially in combination with other innovative concepts) and its potential benefits for not only residents but also staff members and stakeholders are missing.

In contrast, theoretical developments in nursing have taken place on a more fundamental level. In 1997, the Pioneer Network was founded to improve the quality and concept of long-term care in the United States, which marked the beginning of the “NH culture change movement”. The movement seeks to renew NH culture based on the understanding that,

“culture change is a process, and as such, the term connotes a transformation of NHs that goes beyond superficial changes to an inevitable reexamination of attitudes and behavior, and a slow and comprehensive set of fundamental reforms. Culture-change proponents aim to create caring communities where both empowered frontline staff and residents can flourish, and where residents experience enhanced quality of life.” [23]

Conceptually, this multifaceted approach aligns with theoretical ideas such as positive gerontology, successful or productive ageing, and “thriving” in NHs [24–26]. They emphasize the health- and life satisfaction-promoting potential of activity and the continuation of personhood and social involvement in late phases of the lifecycle. Challenging the function of NHs as “total institution”, they reimagine them as salutogenetic living environments and new homes for older people [27–29]. Studies show that culture change practices in NHs (e.g., patient-centeredness, staff empowerment, physical environment improvements) might reduce health-related and quality of life-related deficiencies or prevent an increase of such shortcomings [30].

In this paper, we present the results of a qualitative study on a non-profit NH located in Western Germany whose care approach is based on “therapeutic care practices with rehabilitative elements” [31, 32]. This concept had been developed in this NH over 20 years and combines therapeutic care practices with values similar to the NH culture change movement. The NH at the center of this study became known to insurers as data identified that, following the receipt of short term care, many residents were able to return to independent living in their own home. To understand the care practices including what enabled the improved health situation, we analyzed this NH in order to develop a potential best practice model for therapeutic care in German NHs. Our analysis of the care concept, its practices and effects resulted in the development of a theory of change (ToC) model. In this article, we ask how older people can remain mobile and self-sufficient for as long as possible (any potentially return to their own home).

## Methods

The study was conducted from February to July 2021 with an ethics approval from the University of Potsdam (16/2021). All data on the NHs and German healthcare system was gathered and analyzed in German. For this article, the final results were translated into English by the qualitative research team (AD, CH) and checked for appropriateness by the co-authors. In case of special nursing terms, the German word was added after the translation for transparency.

In order to make our analysis more transparent we relied on three guidelines for developing and reporting a ToC model. We chose several guidelines to ensure that we cover relevant elements while also benefiting from some complementing aspects they provide (e.g., ceiling of accountability). As we developed the ToC model retrospectively, not all characteristics were applicable to our study (e.g., extended stakeholder workshops). We used the Checklist for reporting ToC in Public Health Interventions, the Enhanced Medical Research Council’s (MRC) framework for complex interventions, and the Ecosystem Services for Poverty Alleviation (ESPA) guide to working with Theory of Change for research projects [33–36].

### Theory of change methodology

The theory of change approach provides a methodology to develop, implement, and evaluate complex interventions. It is a theory-based research tool for a contextualized description of an intervention’s effects in its causal relationships, thus, explaining why and how an intervention works [33, 36]. ToC differentiates between measurable short-term, intermediate, and long-term outcomes and, thereby, acknowledges the causalities of activities and different stage outcomes, including adverse effects and nonlinear pathways [33, 35–37]. It considers the contextual conditions, rationales, and assumptions under which the intervention is to operate in the intended way [33, 36, 37].

According to Breuer, Lee, De Silva and Lund, there are four essential elements of a ToC approach [36]. These are the definition of the desired long-term changes, the description of the process or sequence of change, the elaboration of relevant contextual factors and conditions, and the critical reflection on assumptions on which the ToC is based on.

De Silva, Lee and Ryan enhance the MRC framework for complex interventions and define essential ToC elements which should be developed backwards from the intended social impact and in collaboration with relevant stakeholders [34]. They recommend five steps for developing a ToC model. First, define the real-world impact that is aimed at; second, decide on the long-term and intermediate outcomes and define the causal pathways between them; third, add the necessary interventions or activities required to achieve these outcomes; fourth, add assumptions or rationales to the causal pathways; fifth, define indicators of success for each outcome. Additionally, a ceiling of accountability should be drawn, which separates the measurable outcomes for which an intervention can be directly responsible from the broader social impact.

The ESPA ToC manual equivalently covers these elements in three steps (defining long-term change or impact, sequencing of events, making assumptions explicit) [35]. While it does not explicitly mention a ceiling of accountability, it highlights an important step prior to the impact definition. The author suggests a baseline context analysis where the underlying problem, relevant actors and power relations as well as the context’s receptiveness to change should be discussed [35].

### Data collection and material

We incorporated four different data sources for qualitative analysis. First, we conducted an online search for written or visual material about the NH under investigation, which is located in a rural area in Western Germany. This included publicly available information, i.e., their website, ten newsletters from the years 2020–2021, seven promotional videos produced in collaboration with a statutory health insurer or public service television network, and two newspaper articles written by the facility’s director. This material provided information about the core elements of the therapeutic nursing concept, logistical characteristics, the intended impact, and concrete practice examples of nursing and interaction with relatives. We also collected anonymized nurse records on residents’ curricula vitae or nursing and social care documentation, which gave us insights in the NH’s information management and focus on individual life history.

Next, we conducted 16 qualitative interviews with 14 staff members from the innovative NH who work in different positions (nurses, social care workers, housekeeping personnel, therapists, a physician; the nursing director and his assistant were each interviewed twice). Participants were purposively sampled based on key issues emerging from the document analysis including the interprofessional teamwork, interactions between staff, relatives, and regional community, and the special focus on therapeutic elements and individual biography in the nursing practice. An invitation to participate in this study was forwarded by the NH director, who recruited staff from various positions. Those, who agreed to be interviewed contacted the interviewer in order to schedule an interview. Interviews took place via telephone or video call and ranged between 45 and 90 min. After an opening question about the benefits and challenges of the therapeutic care concept, the interview guideline covered topics such as the interviewee’s particular role, necessary qualifications, collaboration with other staff members, interactions with residents and relatives, and concrete practice examples. To contrast the concept and practices identified from the NH’s study participants as well as to further and finalize the best practice model, we conducted ten interviews with NH directors and care managers from ten other NHs in rural and urban areas in the northern and northwestern parts of Germany. The NHs were previously contacted by a statutory health insurance for a possible implementation of therapeutic nursing practice based on the studied NH in the future. The selection criteria for these NHs were a location in the insurance’s region of responsibility, a balance of NHs in rural and urban areas, the inclusion of public, private and non-profit NHs, and an interest of the NH management to test an innovative nursing concept. Interviews were conducted as telephone or video calls and lasted between 20 and 45 min. This interview guideline covered a presentation of potential core elements of the developed best practice model and comparing question in terms of practices, values, infrastructural conditions, residents, and staff in the respective NHs. All 26 interviews were audio-recorded, protocolled, and pseudonymized for the analysis.

We then reviewed literature on the German healthcare system and nursing home challenges to provide contextual support on the explanations given by study participants and stakeholders. Our aim here was to provide further clarity for international readers regarding the “taken-for-granted” or “tacit” knowledge on the problems and issues in Germany which is grounded in sociocultural experience and shared by study participants and researchers to varying degrees [38].

And forth, we used international literature on concepts and practice examples of therapeutic and rehabilitative care, ToC methodology, and NH culture change to discuss the results.

**Fig. 1:**
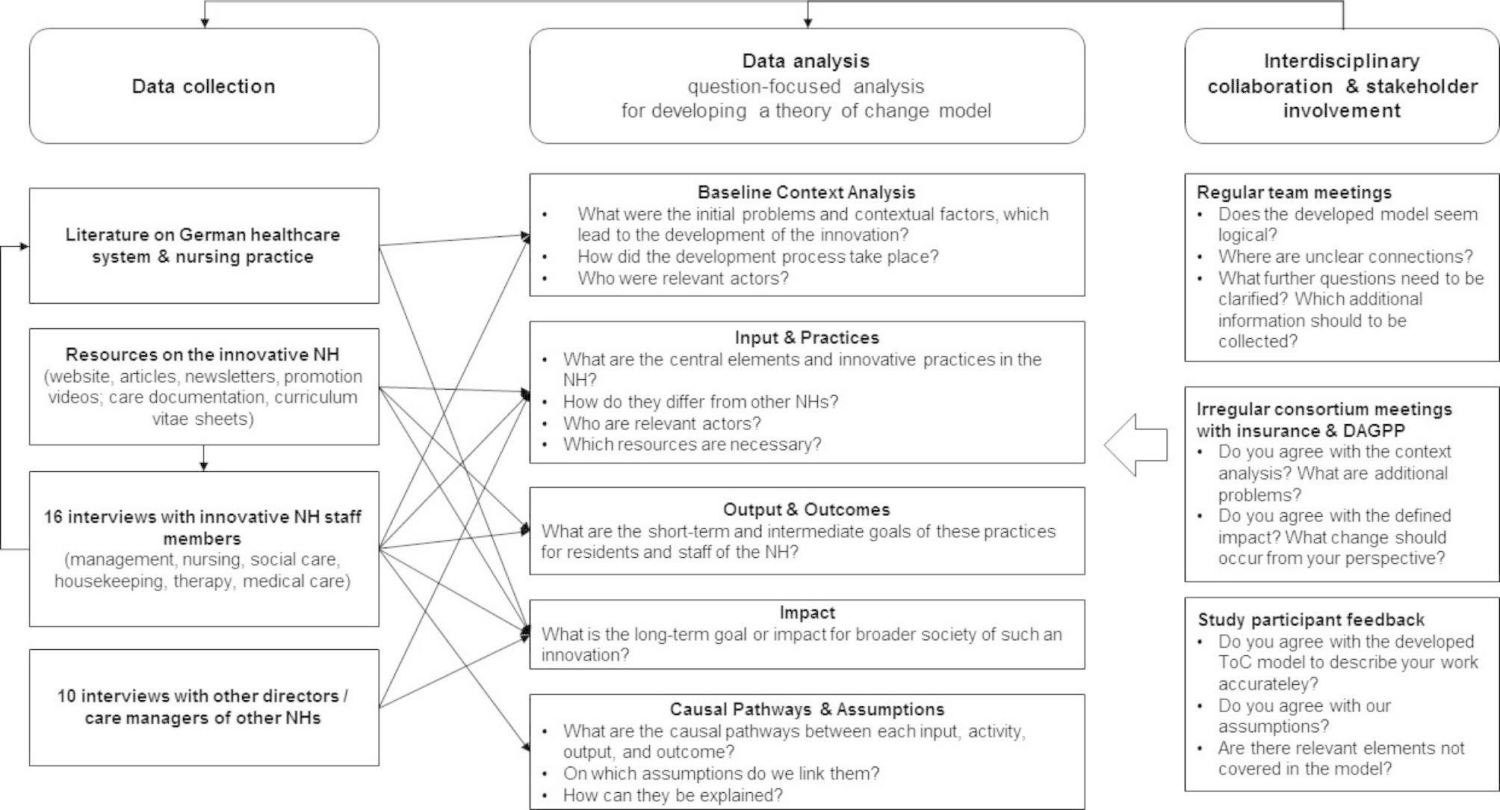
Data collection, question-focused analysis, and collaboration in the ToC development process

Based on the ethnographic research tradition, the initial data collection plan included observations of care practices and team collaboration along with staff interviews in the innovative NH [39]. However, due to the Covid-19 pandemic, observation via field visits in the participating NH could not be realized. Instead, we relied on the document analysis and qualitative interviews with staff members of the NH, supported by contextual literature to gain a complex and contextualized understanding of relevant components for the ToC development. Each kind of material provided information on specific aspects which could be supported or complemented by other forms of data (see Fig. 1). An openness to a multimethod approach should reduce the risk of missing important aspects of the NH’s concept [40]. The data set was not pre-set, but rather built in an inductive, iterative pathway whereby collected material and ongoing analysis informed the gathering of further information.

### Data analysis

While the ToC approach was developed as an intervention planning and evaluation tool, we applied it as an analytical lens to help uncover the components and effects of an already existing innovative NH of therapeutic care for older people living in a NH in Germany. We retrospectively developed a ToC model covering the essential elements defined in the guidelines [33, 35, 36]. To do so, we analyzed all material in an iterative process guided by the questions depicted in Fig. 1 and subsequently organized it in a ToC model.

### Interdisciplinary collaboration and stakeholder involvement

The project’s consortium was comprised of a psychiatrist, two psychologists, two anthropologists, a biostatistician, two health economists of a statutory health insurance (AOK), the German Academy for Gerontopsychiatry and Psychotherapy (DAGPP), and the management team of the innovative NH. Interdisciplinary collaboration took place in weekly online team meetings between the core team comprised of the psychiatrist, psychologists, and anthropologists, where results from the qualitative interviews and the current state of the ToC were discussed.

To include additional stakeholders, larger meetings with the health insurance and DAGPP were held in irregular intervals. The research team presented explanations of contextual factors, problems, and preferable change which were given by interviewees. Similar to DELPHI rounds, participating stakeholder members discussed and agreed on the relevance of the issues provided by NH staff [41]. Additional questions enhanced the information on contextual factors, especially concerning health and nursing legislation.

The results and the developed ToC model were communicated to all interviewees of the innovative NH. We received agreement via email from participants to have covered all relevant aspects in the ToC model.

## Results

**Fig. 2:**
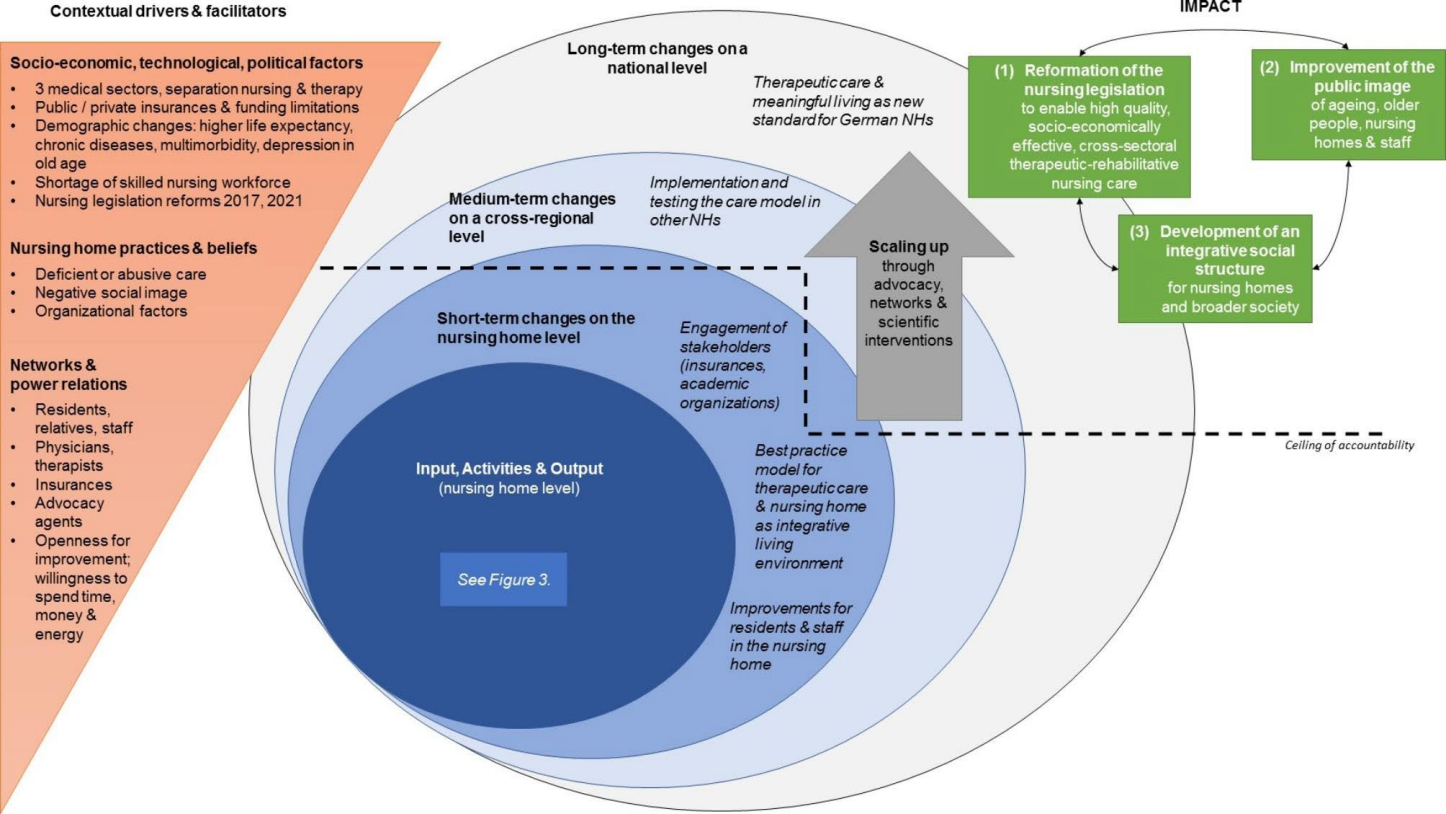
Theory of change model on a national level (based on the ESPA ToC guide) [35]

**Fig. 3:**
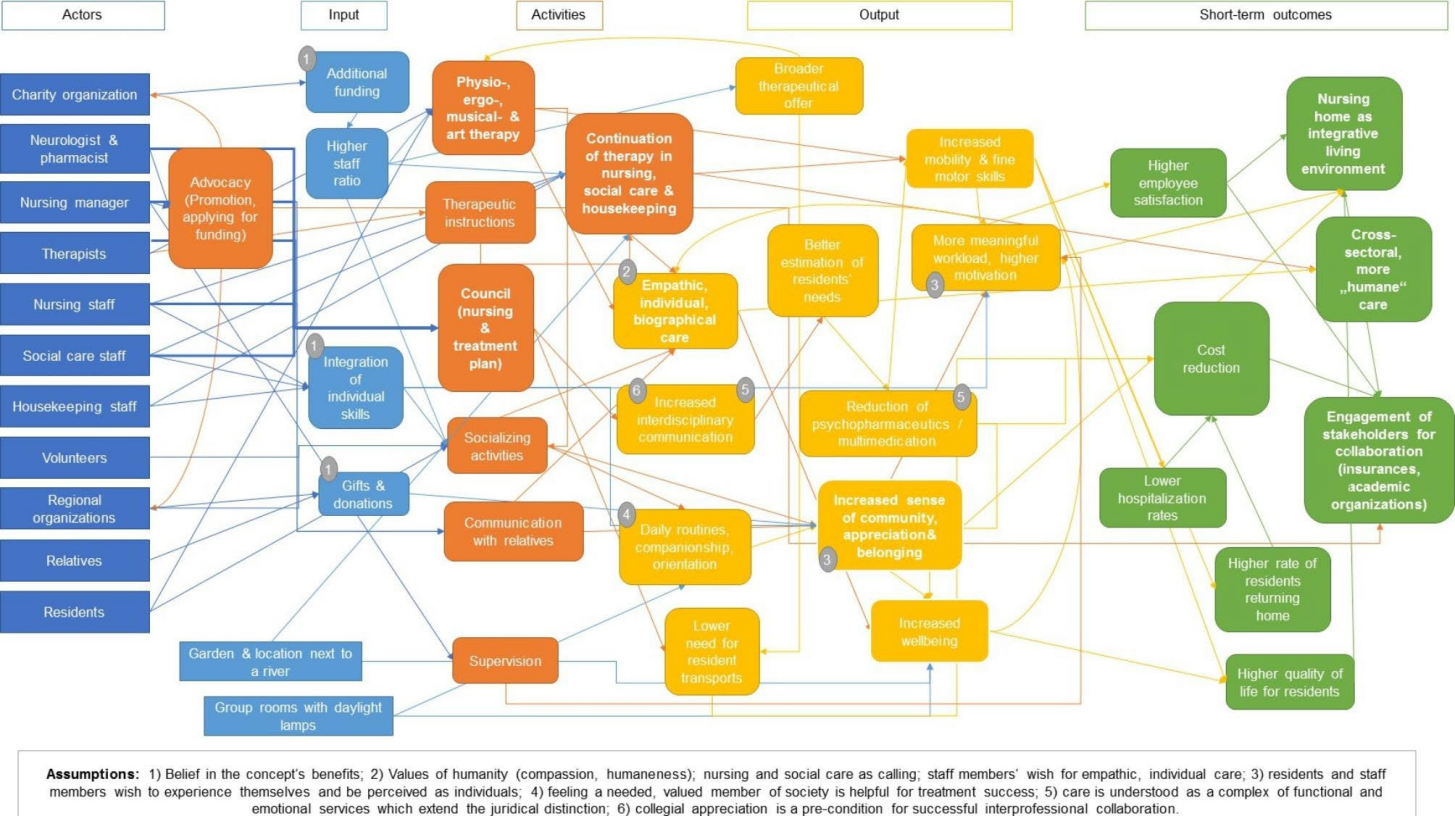
Theory of change model on the participating NH’s level

The overall ToC visualization in Fig. 2 shows the contextual drivers and facilitators as well as the intended medium- and long-term social impact of a possible transfer of the innovative NH’s concept to other NHs and finally on a national level. Fig. 3 displays the concrete input, activities, output, short-term outcomes, causal pathways, and assumptions on the local level.

### Contextual drivers and facilitators

Different challenges on a broader societal level as well as problems experienced in nursing practice were described by our interview partners and stakeholders as impulses for change. They are related to structures of the German healthcare system, nursing legislations, and demographic developments which are reflected in recent literature.

#### Social, economic, and political conditions

In the German statutory health care system, services are historically divided into three different sectors with specific tasks, remuneration systems, accessibility, and referral procedures [42]. The three sectors are outpatient health care, hospitals, and outpatient or inpatient rehabilitation. This structure poses challenges to patients, practitioners, health institutions, and national funding. It entails a high level of bureaucracy, economic burdens for insurances due to ineffective use of services, and suboptimal interprofessional collaboration and patient pathways [43]. The majority of the population (87.8%) is publicly insured [44]. While private health insurance terms vary, they tend to refund more than the statutory ones. Less established treatments, e.g., musical therapy, are not covered by public health insurance. Similar differences exist between statutory and private nursing care insurances, which pay for nursing care at home and in NHs.

According to German legislations, therapeutic treatment shall not be provided by nursing or social care staff but only by therapists and needs to be prescribed by physicians. Likewise, basic nursing activities (“Grundpflege”, i.e., personal hygiene, eating, mobility, prevention, self-sufficiency, and communication) shall only be carried out by nurses, not by social care workers. This legal distinction between therapy, nursing, and social care sometimes conflicts with the reality of interactions between staff and residents, as will be shown below.

Because of the highly demanding working conditions, fewer qualified nurses work long-term in professional care settings. Frequent sickness, early retirement, and change of profession are common phenomena [45]. In light of the shortage of skilled labor in the care sector and the continuously growing number of people in need for care, Germany seeks to develop innovative approaches. One of them is to reduce stationary care, guided by slogans such as “rehabilitation before care” and “ambulant before stationary” [1]. Recent strategies to substitute workforce losses with qualified nurses from other countries have not been sufficiently effective [46]. To improve working conditions, the nursing legislation reform from 2017 redefined the nurse-per-resident-ratio for each federal state in accordance with residents’ care need levels. However, the Covid-19 pandemic has demonstrated and worsened the problematic shortage of skilled nursing staff as even more care workers leave their profession [47]. A new Nursing Reformation Act was passed in 2021, by which agreed wages for nurses shall be raised and a nation-wide staff ratio implemented by 2022/23 to counteract the current problematic developments.

#### Residents’ and staff’s experiences in nursing homes

The innovative NH’s concept had been developed over a timeframe of 20 years by the NH director, staff members, and other collaborating actors. A key motivation were the perceived “inhumane” conditions in the NH and broader medical practices. Physicians considered many older people as “not mobilizable”. Indeed, participants complained that residents were often confined to a wheelchair and positioned in front of the television all day. Further, immobility of residents was induced or worsened by polymedication or as adverse effects of medications, such as sedatives for dementia [48]. Additionally, depression or embitterment disorder (“Verbitterungsstörung”) due to the experience of a traumatic life event or gradual decrease in quality of life may reduce older people’s motivation and capacity to engage in physical activities and vice versa [49]. In such cases, psychosocial therapies are a necessary component to improve mobility, but they are not provided as needed. A study from 2019 showed that only 12% of older people experiencing depression are receiving psychotherapy in Germany [50].

Neglect of older residents in institutional settings is a worldwide phenomenon [51]. According to studies, it has an occurrence of 11 to 14% in Germany [52]. Many cases of deficient or abusive care in German NHs depict negative images of NHs as “last stop” or “custody care” [53–56]. They illustrate a range of interconnected problems such as the shortage of qualified nurses, a lack of quality and empathy in care, an overuse of neuroleptics and excessive restrictions of residents’ autonomy, undernourishment, and insufficient therapeutic and social activity offers.

Elder mistreatment is associated with different organizational aspects (e.g., infrastructural deficiencies, poor management, unskilled or unmotivated staff, resident characteristics, institutional work culture) as well as macro-structural factors [7]. This includes bad working conditions for NH staff such as a high work load, interpersonal conflicts, poor salary, lack of appreciation for their work, and excessive documentation requirements [57]. Nursing staff frequently experiences stress-related health issues (e.g., sleeping problems, depression and burnout, musculoskeletal disorders) and engages in health-damaging behavior such as smoking, poor diet, or substance abuse [58]. Additionally, many nurses are frustrated about not being able to care for residents as they would like to, which compelled the NH director to initiate a change of practice.

#### Networks, infrastructure, and power relations

NHs in Germany do not usually employ their own physicians or therapists. As patients have freedom of choice, residents in NHs could theoretically select their preferred physician or specialists. Upon moving into a NH, many residents prefer to keep their former physician. However, working with a NH requires physicians and therapists to have enough time for regular visits, or residents to be transported to the physician’s or therapist’s practice. To reduce these costs, NHs try to form cooperation contracts with physicians, specialists (e.g., cardiologists), dentist, and therapists. In some cases, however, there are not enough physicians and therapists available to form such a cooperation, especially in rural areas. Interactions between physicians, therapists and nursing staff usually occur via medical and nursing documentation, prescriptions, and referrals. According to our interview partners, communication via fax is still a common practice in many NHs as physicians are too busy to be available for telephone calls and email is not well established.

In the case of the participating NH, a collaboration was formed between the NH, a neurologist/psychiatrist, and a pharmacist. This alliance developed through a long-term exchange about the problems inherent to NHs and a shared vision of an ideal quality care. The key actors (NH director, neurologist, and pharmacist) promoted and advertised the NH’s approach to form collaborations and gain funding for additional therapeutic, nursing and social care activities. Recently, articles about the NH have been published to further promote the concept [31, 32]. However, the approach was met with resistance by insurers and policymakers in the past, who argued that such a nursing concept was too expensive, not feasible, and working against the legal distinction between medical treatment and nursing practice. Encouraging such a project was even considered as “opening Pandora’s box” with financially unsustainable and large-scale political consequences. Shifts in the stakeholders’ management boards and the persisting problems in nursing care contributed to a change of perspective and a collaboration between a large statutory health insurer and the NH after several years.

### Input, activities, and outcomes on the nursing home level

As depicted in the theory of change model in Fig. 3, input, activities and outcomes do not necessarily succeed each other in chronological order, but were established in iterative interactions over time.

Relevant *actors* in and outside the innovative NH are a charity organization, a neurologist, a pharmacist, the NH director, therapists, nurses, social care workers, housekeeping staff, volunteers, regional organizations (e.g., the local fire department, a regional orchestra), residents themselves, and their relatives. Additional *input and resources* are extra funding from a charity organization enabling a higher staff ratio, the integration of staff members’ individual skills, gifts and donations from organizations and relatives, the facility’s location next to a river and an adjacent garden, and its living and group rooms with daylight lamps. Compared to other facilities of similar scale, the NH employs additional 7.5 full-time positions in nursing, 1.2 in therapy, and 1.0 in care management. The overall staff-to-resident ratio is 1:2 and therefore much higher than legally required. This allows for additional therapy and social activities and more time for the therapeutic nursing practice itself. Innovative *practices* of the NH include enhanced interprofessional team meetings, therapeutic instructions to nurses, a broader therapy and social activity offer, systematic continuation of therapy in nursing and social care, advocacy for the therapeutic care concept, supervision of staff members, and increased communication with relatives. The practices lead to numerous, mutually reinforcing effects. These *mechanisms* are described in detail in the following paragraphs. Possible *measurable outcomes* of the activities’ effects could include a higher employee satisfaction, higher quality of life for residents, cost reduction for insurances, and stronger engagement of regional stakeholders.

In the monthly case meetings, primary nurses (“Bezugs-pflege”), housekeeping, and social care staff meet up with therapists, a neurologist, and a pharmacist to define and monitor each resident’s therapy and rehabilitation plan. Case meetings in other NHs do not include as many different professions, especially missing regular participation of a neurologist, pharmacist, and therapists. These enhanced case meetings are crucial because all different professions come together as a team and think about treatment potential from their respective expertise. This enables a more comprehensive estimation of the residents’ state and needs. Neurologist and pharmacist reconsider the medication, especially regarding maleficent polymedication and adverse effects (e.g., decreased mobility). Therapists suggest treatment options and potential for rehabilitation. Primary nurses and social care staff provide first-hand knowledge about the resident’s needs, individual life history, possible motivation for engaging in activities, and treatment progress.

Informal communication between colleagues is highly relevant as well. During shift changeovers or regular care practices, information about residents’ daily situation and treatment progress is exchanged. Staff members ask others for help if they lack the required knowledge, time, or practical qualification for the resident’s needs, which makes the provided care more effective.

The NH calls their own approach “therapeutic care with rehabilitative elements”. The central aspect, therefore, is the integration of therapeutic measures by (social care) nurses into everyday activities. This process is accompanied by the multidisciplinary team meetings and rehabilitative goalsetting described above. In practice, therapeutic care means that housekeeping, nurses, and social staff encourage and assist residents in managing tasks themselves. Thus, they promote self-sufficiency and attend to each resident’s needs, capacities, and rehabilitation potential individually. For example, residents are helped into their shirt but may be encouraged to button it themselves. Similarly, encouraging the use of the hemiplegic arm training was shown to take place not only during therapy sessions but also during activities of daily living (ADLs), such as eating and grooming.

To enable nursing and social care staff to include such therapeutic elements, therapist regularly instruct other staff members on exercises to be integrated in ADLs. For example, nurses are advised to avoid using hoists and instead try to assist residents to transfer or use a mobility frame in order to promote standing and walking endurance during daily routines. Physical and ergonomic therapy and its continuation in nursing care was perceived to lower the need for residents to be transported to the session with external therapists and physicians, and to increase their mobility and basic life skills. Preliminary quantitative analyses by one of our team’s primary researchers (MR) showed reduced rates of hospitalization and higher rates of residents returning to their own home. Both aspects together with lower medication rates may contribute to a cost reduction for insurers.

However, the broader therapeutic offer to residents consists not only of physical and ergonomic therapy but additional opportunities for stimulation and physical mobilization through musical and art therapy which is financed by a charity organization. Music and art are constantly integrated into everyday activities (e.g., while getting up, during meals) to stimulate and activate residents. As the NH’s musical therapist described it, music has the capacity to connect different people in a joint activity and is especially useful to activate memories, revive life history, and help residents to feel a sense of identity. In some instances, however, musical or art therapy might evoke painful memories that need to be worked through with residents, therapist, nurses, and sometimes relatives.

Residents’ treatment successes contribute significantly to staff members’ professional satisfaction and work motivation. Staff members view their work as more meaningful which, in turn, fosters greater empathy and care underpinned by a focus on residents’ individual histories.

Social activity offers (e.g., bowling, newspaper reading, singing together, conversation, gaming afternoons) provide leisure activities, communication, and a sense of community among residents. They are offered by social care workers, volunteers, and sometimes relatives. Special social activities such as concerts are held by regional organizations and help residents to feel connected to the broader society while enjoying entertainment. Apart from the availability of activity, social care workers provide meaningful companionship and a daily structure which are crucial to residents’ wellbeing. Social care workers accompany residents, stimulate conversations, or simply listen to them. This kind of relationship work is essential for figuring out how to motivate residents and engage them in the therapeutic care process. Such findings are then communicated to other professions through the formal or informal processes described above.

In the innovative NH, the integration of staff members’ individual skills and trainings had been developed organically over time. For example, housekeeping staff might play music at events such as church holiday celebrations. This helps residents to recognize staff members as individuals with different roles and characteristics and increases a sense of familiarity and community. On the other hand, staff members feel appreciated as individuals and can participate in different activities in the NH, which increases their job satisfaction.

Regional organizations and relatives contribute by sending gifts and donations like flower arrangements for residents or homemade cake for staff members, which helps to create a sense of community, appreciation, and belonging between the NH and local community.

The NH’s garden and the facility’s location next to a river serve as additional spaces for therapy, leisure activities, and relaxation, and therefore support the daily routines as well as residents’ wellbeing. Similarly, large living rooms with integrated daylight lamps add to a balanced day-and-night-rhythm, promote a comfortable atmosphere, and provide space for encounters with staff and other residents.

While some of the other regional NHs follow aspects of therapeutic care or other NH innovations (e.g., activating care, leisure activities, animal-assisted therapy), none of them applies them as systematically and comprehensive as the innovative NH. Although the concept and practice of therapeutic care has been refined over the past 20 years, occasional problems arise. Shortage of staff is still an issue because individual, therapeutic care requires more time. The NH director supervises the execution of therapeutic care, shortcomings are discussed, and improvement strategies developed in collaboration with other staff members.

### Long-term impact on the national level

The intended long-term impact of all these activities covered interrelated aspects of the problems in nursing care (see Fig. 2). As a final aim of our broader society, a *reformation of the nursing legislation*, we posit the need for high quality and socio-economically effective nursing practice that works across sectors and includes therapeutic and rehabilitative elements. This should entail financial compensation and higher staff ratios that improve working conditions and therefore the quality of care, residents’ care experience, and staff’s physical and emotional wellbeing. Such a legislation could ultimately make the nursing profession itself more appealing and thus help to reduce the nursing staff shortage.

The second aim concerns the *improvement of the public image* of ageing, older people, NHs, and staff. More precisely, this is accompanied by an appreciation of old age as a valuable stage of life and older people as valuable members of society. NHs should be viewed and experienced as a living environment based on values of humanity for residents and staff alike. Nursing and social care staff should be recognized as crucial actors for creating such a living environment. The willingness of stakeholders and policy makers to increase funding and reshape legislation should grow through this heightened appreciation.

Third, an *integrative social structure* should be promoted by including older people in NHs into the broader society’s activities and collaborations with regional actors and stakeholders.

### Assumptions and rationales

As a first precondition for the NH’s effectiveness, relevant actors needed to be convinced of or open to the innovation’s potential benefits. This is crucial for the NH’s staff as well as medical and pharmaceutical experts, charities and stakeholders as potential funders, and regional organizations.

The ToC’s underlying mechanisms and assumptions are connected to the organizational culture that is established in everyday interactions. A second assumption, therefore, is that NH practices are based on values of humanity, such as compassion, humaneness, or grace. Staff members are then more likely to care for residents in an empathic, individual way, assuming they have enough time to do so. Nursing, as the innovative NH’s employees described, is considered a calling, not just a job. However, not all nurses have the same work ethic or competences. More empathic nurses should be assigned to work directly with residents while others might be better qualified for organizational tasks, as the innovative NH director explained. Staff members, therefore, need to be considered and assigned to tasks appropriate for their individual strengths and weaknesses.

A third assumption is that residents would like to experience themselves and be perceived as individuals with specific characteristics and life histories. This requires them to open up and trust which can only be reached by sensitive interaction over time and equally applies to staff members who need to feel treated with respect and empathy themselves. Fourth, the experience of being relevant, or needed by others, and a part of the community is essential for residents’ wellbeing, treatment success, and ultimately the creation of a meaningful living environment.

Fifth, care itself is conceptualized in a complex way, which blurs the distinction between nursing and social care enshrined in the German social security legislation. The assumption is that everyone interacting with the residents is taking part in their care, be it the facility manager changing light bulbs and having a chat with them or the neurologist checking their medication. And sixth, as the therapeutic care is heavily relying on formal and informal communication between staff members, mutual appreciation and respect for each other’s expertise are crucial to promote interprofessional learning without competitive thinking between colleagues.

## Discussion

The analysis of the NH’s care concepts allowed us to identify core elements for a best practice model of therapeutic care which helps residents to engage in daily activities in such ways that they are mobilized and care needs reduced. The innovative NH manages to increase residents’ mobility and self-sufficiency by combined measures all of which work together to engage residents in meaningful ways. This includes extended interdisciplinary collaboration for defining and monitoring rehabilitation goals addressed by care staff but housekeeping as well. Mobility is further improved by a broader therapeutic and social activity offer and the integration of therapeutic elements into everyday nursing, social care, and housekeeping practice. Special attention is given to mobility-reducing effects of multimedication and depression. It is also mirrored in the way social spaces of the NH are assembled, including communal areas, to include music and movement into daily practices. These spaces provide a piano for playing music and other creative activities that aim to touch the residents emotionally. It is also mirrored in the way all staff is included in providing an environment for meaningful interactions.

For stakeholders such as the involved insurance, the ToC suggests that the reduction of medication, resident transports to physicians and therapists, and hospitalization rates might lower costs. However, these aspects should be further explored through quantitative evaluation methods as this was not the focus of the best practice model. The increased teamwork, empathic care for the resident’s individual needs and life history, meaningful social activities, daily routine, continuity of primary caregivers, and regular interactions with the local community establish the NH as a humane and integrative living environment for both residents and staff members.

Van den Bosch and Rotmans argue that change needs to occur in three domains: structure, practices, and culture [59]. The development of a ToC helped uncover the changes that therapeutic care accomplished in these domains in the innovative NH. Cultural change in NHs, can improve quality indicators for provided and perceived care [30, 60]. The underlying assumptions of the ToC illuminate the change of NH culture that was necessary to establish the NH’s approach. They include commonly shared values of humanity and empathy, a recognition of residents’ and staff members’ individual life histories and capacities, their need for social inclusion, and a complex understanding of the practical and emotional nuances of caregiving.

Partially, these innovative elements have been successfully applied in different international settings. Many health-promoting projects aim at improving NH residents’ mobility or show positive effects of musical therapy on residents with psychological illnesses such as dementia or depression [61–64]. A focus on individuality and interdisciplinarity seems to be of international importance for nursing practice and social inclusion can have a positive effect on residents’ health outcomes [65–68]. A diversity of tasks, appreciation (e.g., fair wages and social recognition), experiencing one’s work as positive and meaningful, shared leadership, autonomy and participation, and self-care abilities help to reduce nursing staff’s work stressors [58]. Environment-focused innovations, green care homes or horticultural therapy, for instance, showed a positive effect on resident’s perceived quality of life [69, 70].

Likewise, the innovative NH creates a health-promoting physical environment, a salutogenic space which helps form a sense of coherence and cope with or improve older people’s experience of ill-health and social disintegration [71]. Such an environment is fundamentally different from earlier models of NHs as “custody care” facilities or hospitals [72]. Instead, a positive living environment can be formed by “de-institutionalizing” NHs and promoting individuality, companionship, social relations, positive atmosphere, and person-centered care [73].

However, not only changes in physical space, but the work of medical, therapeutic, nursing, social care, and housekeeping staff is crucial for assisting residents in the process of meaning-making in their new living environment. New residents often experience multimorbidities and impaired functionality, social and spiritual disintegration, and the (partial) loss of identity and life purpose [74]. The activation impulses through therapeutic care motivate residents to engage in ADLs and thereby partly re-locate the “locus of control” back to residents [75]. Nursing activities do not happen to or for them, but become a shared responsibility between staff and residents.

In addition to high quality medical care, residents are in need of rebuilding a coherent and meaningful story for their experience. Through what Mattingly termed “therapeutic emplotment”, residents and the multiprofessional team bring their own experience of people, time, and space into a shared meaningful narrative structure [76]. The daily structure provided by nursing and social care staff along with individual or group therapies and social activities function as interpretative acts whereby residents’ stories and identities are rebuilt. Consequently, they are able to regain a sense of coherence and meaningfulness. Not only do they establish a “plot” for their life in the NH by following a meaningful daily structure. Especially art and musical therapy, the focus on biographical events, and interactions with the community outside the NH contribute to connecting residents’ lives before and after their relocation to the NH. Others have shown how these interpretative acts of meaning-making take place during doctor-patient-interactions or through interprofessional collaboration [76, 77]. In the innovative NH, the therapeutic emplotment is an essential part of their therapeutic care concept and practiced in every aspect of residents’ lives. Artioli, Foà and Taffurelli describe these practices on a conceptual level as combining evidence-based medicine with narrative-based nursing models for a bio-psycho-social approach to an “integrated narrative nursing” and call for best practice examples in nursing [78].

We developed a best practice model based on the participating NH which systematically combines different elements of innovative geriatric nursing practice. Similar to the concept of APNs, staff members in the studied NH take on more responsibilities. Provided that they are appropriately trained, more responsibility for nurses can make the nursing profession more attractive and compensate for the shortage of physicians, especially in rural areas. Moving beyond activating care, the therapeutic and rehabilitative care can not only take place in special rehabilitation clinics but in NHs in general. A cultural change focusing on rehabilitation through individuality and social integration establishes the studied NH as a partly de-institutionalized positive living environment for therapeutic emplotment between residents and staff.

One limitation of our study is that we were not able to do field visits in the NHs but had to rely on the descriptions given by our interviewees. Observations could have shown potential gaps between the therapeutic care theory and actual practices [39]. Also, our interview partners at the NH were contacted by the NH management. Thus, we may have a very selective interview sample that fit particularly well with the promoted concepts of therapeutic care by the NH. We tried to reduce this shortcoming by including other material such as the nursing documentation and discussing problems and possible improvements with interviewees. It is important to note that the developed ToC model is a first attempt to systematically describe the mechanisms of the NH innovation to develop a best practice model which needs to be tested by further research. Further qualitative research could include observations of practice examples as well as interviews with residents and relatives. A quantitative evaluation of resident, staff, and stakeholder outcomes (e.g., quality of life, work satisfaction, insurance costs vs. additional funding) could falsify the assumptions made in the ToC model.

Due to the retrospective application of the ToC methodology, the involvement of stakeholders was less prominent than usually required by guidelines. Stakeholder involvement is crucial for defining the desired impact and characterizing the context and need for change [33–36]. However, for applying the ToC methodology to already existing innovations where intended outcomes are already determined, adapted guidelines might be useful. The process of scaling-up the NH’s innovation horizontally (i.e., implementing and testing it in other NHs) as well as vertically (i.e., implementing it as a national standard for German NHs and developing the necessary legislations) is beyond the scope of this study and would require extensive research and stakeholder involvement [79].

## Conclusion

Older people’s functionality is highly relevant for their self-sufficiency and social integration. The ToC shows how residents’ mobility can be strengthened through interprofessionally supported therapeutic care that is adapted by all staff who are in contact with the residents of a NH. A meaningful living environment for NH residents is created by music, art, and social activities, and sufficient time for therapeutic practices in daily interactions. Encounters between staff members and residents work as narrative emplotments through engaging and motivating residents by providing frameworks and daily goals. Staff members, thus, can spark hope in oftentimes depressed older people that their future time in the NH is something to look forward to and engaging in therapy is meaningful and important for their wellbeing. This highlights the significance of meaning-making as a central aspect of nursing and social care, which should be considered just as much as improving residents’ functionality.

Appropriate political frameworks and infrastructures need to be shaped to implement innovative nursing practices of therapeutic care in NHs. Underlying cultural values and necessary changes in the appreciation of the older and the nursing profession should be acknowledged and guide concrete political changes. Consequently, contextual factors and organizational culture should be considered in intervention development and implementation. The goalsetting of health and nursing interventions should be further expanded to incorporate psychosocial aims next to biomedical ones and recognize their interrelatedness. Innovations should also make use of previous research on shared narrative constructions in care interactions and its therapeutic effect in the healing and care process.

We benefited from the complex ToC methodology by uncovering the “black box” of the nursing concept’s underlying mechanisms and recognizing contextual conditions. As the ToC approach is intended for embedding interventions in the “real world”, we consider it a promising tool to analytically uncover existing innovative practices. Using the ToC approach not only helps to understand the interrelations between the components of the NH’s therapeutic nursing practice and social integration, but it might also promote international comparison and adaptation into different settings through vertical or horizontal scale-ups.

## Data Availability

The datasets generated and/or analyzed during the current study are not publicly available due to participants’ rights of privacy and data protection but are available from the corresponding author on reasonable request.

## Abbreviations

NH: nursing home
BPM: best practice model
APN: advanced practice nurse
NP: nurse practitioner
ToC: theory of change
MRC: Medical Research Council
DAGPP: German Academy for Gerontopsychiatry and Psychotherapy
ESPA: Ecosystem Services for Poverty Alleviation
NASHIP: National Association of Statutory Health Insurance Physicians
KBV: Kassenärztliche Bundesvereinigung
MDS: Medical Service of the National Association of Statutory Health Insurance Funds / Medizinischer Dienst des Spitzenverbandes Bund der Krankenkassen
ADLs: activities of daily living
